# Nitric oxide deficiency and endothelial–mesenchymal transition of pulmonary endothelium in the progression of 4T1 metastatic breast cancer in mice

**DOI:** 10.1186/s13058-018-1013-z

**Published:** 2018-08-03

**Authors:** Marta Smeda, Anna Kieronska, Mateusz G. Adamski, Bartosz Proniewski, Magdalena Sternak, Tasnim Mohaissen, Kamil Przyborowski, Katarzyna Derszniak, Dawid Kaczor, Marta Stojak, Elzbieta Buczek, Agnieszka Jasztal, Joanna Wietrzyk, Stefan Chlopicki

**Affiliations:** 1 Jagiellonian Centre for Experimental Therapeutics (JCET), Jagiellonian University, Bobrzynskiego 14 St., 30-348 Krakow, Poland; 2 Department of Experimental Oncology, Hirszfeld Institute of Immunology and Experimental Therapy, Polish Academy of Sciences, Rudolfa Weigla 4 St., 53-114 Wroclaw, Poland; 3 Department of Pharmacology, Jagiellonian University, Medical College, Grzegorzecka 16, 31-531 Krakow, Poland

**Keywords:** Breast cancer, Pulmonary endothelium dysfunction, Endothelial–mesenchymal transition

## Abstract

**Background:**

Mesenchymal transformation of pulmonary endothelial cells contributes to the formation of a metastatic microenvironment, but it is not known whether this precedes or follows early metastasis formation. In the present work, we characterize the development of nitric oxide (NO) deficiency and markers of endothelial–mesenchymal transition (EndMT) in the lung in relation to the progression of 4T1 metastatic breast cancer injected orthotopically in mice.

**Methods:**

NO production, endothelial nitric oxide synthase (eNOS) phosphorylation status, markers of EndMT in the lung, pulmonary endothelium permeability, and platelet activation/reactivity were analyzed in relation to the progression of 4T1 breast cancer metastasis to the lung, as well as to lung tissue remodeling, 1–5 weeks after 4T1 cancer cell inoculation in Balb/c mice.

**Results:**

Phosphorylation of eNOS and NO production in the lungs of 4T1 breast cancer-bearing mice was compromised prior to the development of pulmonary metastasis, and was associated with overexpression of Snail transcription factor in the pulmonary endothelium. These changes developed prior to the mesenchymal phenotypic switch in the lungs evidenced by a decrease in vascular endothelial-cadherin (VE-CAD) and CD31 expression, and the increase in pulmonary endothelial permeability, phenomena which coincided with early pulmonary metastasis. Increased activation of platelets was also detected prior to the early phase of metastasis and persisted to the late phase of metastasis, as evidenced by the higher percentage of unstimulated platelets binding fibrinogen without changes in von Willebrand factor and fibrinogen binding in response to ADP stimulation.

**Conclusions:**

Decreased eNOS activity and phosphorylation resulting in a low NO production state featuring pulmonary endothelial dysfunction was an early event in breast cancer pulmonary metastasis, preceding the onset of its phenotypic switch toward a mesenchymal phenotype (EndMT) evidenced by a decrease in VE-CAD and CD31 expression. The latter coincided with development of the first metastatic nodules in the lungs. These findings suggest that early endothelial dysfunction featured by NO deficiency rather than EndMT, might represent a primary regulatory target to prevent early pulmonary metastasis.

**Electronic supplementary material:**

The online version of this article (10.1186/s13058-018-1013-z) contains supplementary material, which is available to authorized users.

## Background

Breast cancer kills approximately 40,000 people worldwide each year and is a leading cause of cancer death in women [1]. Breast cancer progression is associated with inflammatory responses that promote neoplastic disease and reduce survival of patients regardless of their age, race, tumor stage, and body mass index [2]. Inflammation is both a marker and the causative factor of endothelial dysfunction and promotes cancer growth and metastasis. In particular, adhesion of metastatic cancer cells to the activated vascular endothelium and their subsequent transendothelial migration are regulated by a number of endothelium-dependent mechanisms favoring or inhibiting premetastatic microenvironment formation. Indeed, factors released from dysfunctional endothelium activate some inflammatory signaling pathways in cancer cells, promoting their invasiveness [3, 4], while endothelial vasoprotective mediators including nitric oxide (NO) inhibit adhesiveness of cancer cells to endothelial cells [5].

Decreased NO production, frequently associated with a decreased phosphorylation of eNOS [6], represents the early hallmark of endothelial dysfunction [7]. NO prevents endothelial inflammatory activation [8]. When its production or bioavailability is compromised, expression of cell-surface adhesion molecules on the endothelium surface such as vascular cell adhesion molecule 1 (VCAM-1) is increased [8]. Endothelial dysfunction can also be activated by platelet-released substances that trigger endothelial inflammation. Furthermore, proinflammatory activation of endothelial cells favors monocyte and neutrophil binding as well as platelet rosetting on the leukocyte–endothelial cell surface [9], facilitating anchoring of cancer cells to activated endothelial surface concomitantly with leukocytes forming platelet–tumor cell–leukocyte heteroaggregates [10]. Formation of such aggregates in low-resistance vascular beds, such as in the pulmonary circulation, irrevocably disturbs the laminar blood flow that could further potentiate pathological endothelial activation [11].

Endothelial dysfunction can also be manifested by increased endothelial permeability linked with disassembly of intercellular adherens junction proteins (i.e., VE-cadherin (VE-CAD)) between endothelial cells [12]. An increase in endothelial permeability is the critical event enabling cancer cells to extravasate and form metastases [13–16]. Endothelial permeability is negatively regulated by mechanisms maintaining endothelial barrier integrity, such as the Slit2–ROBO4–ROBO1 signaling pathway [17–19]. Finally, dysfunctional endothelial cells may lose their endothelium-like phenotype via TGF-β-dependent or TGF-β-independent expression of transcription factors such as Snail [20–22], which are reported to suppress endothelium-specific genes [23–25] that initiate endothelial-to-mesenchymal transition (EndMT) [26].

Since there is no comprehensive study characterizing progression of endothelial dysfunction in the metastatic organ from the very early premetastatic phase until the late metastatic phase of the disease, in the present study we aimed to characterize alterations in the phenotype of pulmonary endothelium in relation to the progression of 4T1 metastatic breast cancer injected orthotopically into mice. For that purpose, we measured NO production, eNOS phosphorylation status, markers of EndMT, endothelial permeability, as well as lung tissue remodeling and platelet activation from 1 to 5 weeks after 4T1 cancer cell inoculation into Balb/C mice. We demonstrate that early impairment of NO-dependent function in the lungs precedes the decrease in expression of endothelium-specific proteins indicating an EndMT phenotypic switch, the latter coinciding with the development of early metastatic nodules in the lungs.

## Methods

### Animals

Two hundred and forty female Balb/C mice, 7–11 weeks old, were purchased from Charles River Lab (Germany) and divided into healthy control mice (*n* = 30) injected orthotopically with Hank’s Balanced Salt Solution (HBSS; IIET, Poland) and mice (*n* = 210) injected orthotopically with 1 × 10^4^ 4T1 murine breast cancer cells suspended in HBSS. The mice injected with 4T1 cells were euthanized (ketamine and xylazine, 100 and 10 mg/kg, respectively) in the 1st, 2nd, 3rd, 4th, and 5th week after cancer cell injection. Healthy control mice were euthanized concomitantly with mice in the 5th week of the disease. Throughout the experiment, all animals were housed 5–6 mice per cage, in a temperature-controlled environment (22–25 °C), maintained on a 12-h light/day cycle and given unlimited access to food (AIN; Zoolab, Krakow, Poland) and water. Experimental procedures involving animals were accepted by the First Local Ethical Committee on Animal Testing at Jagiellonian University (Krakow, Poland; permit no. 140/2013) and the Second Local Ethical Committee on Animal Testing in the Institute of Pharmacology, Polish Academy of Sciences (Krakow, Poland; permit no. 41/2017).

### Cell culture

The mouse mammary adenocarcinoma 4T1 cells were obtained from the American Type Culture Collection (ATCC, USA) and were cultured in RPMI 1640-Glutamax medium (Sigma-Aldrich, Poland) supplemented with 10% fetal bovine serum (Gibco, Thermo Fisher Scientific, Poland), 1.0 mM sodium pyruvate (Sigma-Aldrich, Poland), and antibiotic antimycotic solution (100 units/ml penicillin and 100 μg/ml streptomycin, 25 μg/ml amphotericin B) (Sigma-Aldrich, Poland). Cells were cultured at 37 °C in a humidified atmosphere containing 5% CO_2_. For inoculations, only 4T1 cells at the second passage were used. Prior to the transplantations, 4T1 cells were detached using Accutase solution (Sigma-Aldrich, Poland), centrifuged (300 × *g*, 4 °C, 5 min), counted, suspended in Hank’s Balanced Salt Solution (HBSS; IIET, Poland) at the appropriate concentration, and inoculated into the mammary gland of female Balb/C mice. All cell cultures were routinely tested for *Mycoplasma* contamination (MycoAlert Mycoplasma Detection Kit; Lonza).

### Measurement of breast cancer primary tumor and pulmonary metastasis

Body mass was monitored throughout the experiment. To assess the primary tumor growth, the primary tumor volume was measured with calipers each week as described by Kim et al. [27]. After mice euthanasia, primary tumors, lungs, and spleens were excised, weighed, and saved for further analysis. Lungs designated for assessment of metastasis were fixed in formalin and cut into lobes, and the pulmonary metastatic nodules were counted on their surface. After assessment of pulmonary metastasis, lung lobes were paraffin-embedded, cut into 5-μm slices, and stained with hematoxylin and eosin (H&E) to visualize pulmonary metastasis. The lung cross-sections were scanned with a BX51 microscope equipped with the virtual microscopy system dotSlide (objective magnification 20×; Olympus, Japan). To visualize the reorganization of extracellular matrix in the lungs during disease progression, the lung cross-sections were stained with Unna Orcein staining for elastin fibers. Subsequently, randomly chosen visual fields for mice in each experimental group were photographed in such a way that only the lung parenchyma was visible without major pulmonary blood vessels and bronchi. The pictures were subjected to segmentation in Ilastik (developed by the Ilastik team, with partial financial support by the Heidelberg Collaboratory for Image Processing, HHMI Janelia Farm Research Campus and CellNetworks Excellence Cluster), and the relative number of pixels corresponding to elastin fibers in each experimental group was calculated using ImageJ [28].

### Measurement of NO production in the lungs

Colloidal Fe^2+^(DETC)_2_ was used for trapping the intracellular NO with EPR detection as described by Cai et al. [29] with minor changes. Briefly, lungs perfused with ice-cold PBS were excised and cut into small pieces and placed into 0.1 ml Krebs Hepes buffer (NaCl 99 mM, KCl 4.7 mM, CaCl_2_ 2.5 mM, MgSO_4_ 1.2 mM, NaHCO_3_ 25 mM, KH_2_PO_4_ 1.03 mM, glucose 5.6 mM, HEPES 20 mM) on a 24-well plate. The buffer was bubbled for at least 30 min with argon gas on ice to remove oxygen prior to use. Then 2.25 mg of FeSO_4_ × 7H_2_O/10 ml and 3.6 mg of DETC/10 ml were dissolved separately in argon-bubbled buffer, to obtain final concentrations 0.8 mM and 1.6 mM, respectively, mixed, and immediately added to the tissue samples (0.25 ml per well). The tissues were placed in an incubator at 37 °C and incubated for 90 min in an air atmosphere. Tissue samples were then collected, weighed, introduced into 1-ml insulin syringes, and snap-frozen in liquid nitrogen. Measurements of Fe_2_(DETC)_2_-NO signals in frozen samples were performed in a finger Dewar using an EMX Plus Bruker spectrometer with the following settings: microwave power, 10 mW; modulation amplitude, 0.8 mT; scan width, 11.5 mT; scan time, 61.44 s; number of scans, 4. The results were collected, and the amplitude of the characteristic NO triplet spectrum was analyzed using Eleana software.

### Quantitative assessment of Snail expression in the pulmonary circulation

Formalin-fixed and paraffin-embedded lungs were cut into 5-μm slices. Antigen retrieval was performed according to the standard protocol. To visualize expression of Snail, the slices were incubated with primary anti-Snail antibody (ab53519; Abcam) and secondary biotinylated donkey anti-goat antibodies (705-065-147; Jackson ImmunoResearch) concomitantly with ABC vector complex. For each slice, 10 randomly chosen nonobstructed arteries were photographed with a BX51 microscope (objective 20×) equipped with the virtual microscopy system dotSlide (Olympus, Japan) and the length of Snail-positive fragment(s) within the artery was manually measured and expressed as the percentage of the entire circuit of the particular artery. At the same time, the representative images of the investigated arteries were assessed for their patency (i.e., obstruction with blood clot or cancer cells) by a blinded investigator.

### Measurement of pulmonary endothelium permeability by Evans blue

Subsequent to anesthesia (100 mg/kg ketamine + 10 mg/kg xylazine, i.p.), mice were injected via the femoral vein with a solution of Evans blue (EB, 60 kDa) dye (Sigma Aldrich) at a dose of 4 ml/kg. Injected dye solution, composed of 2% EB in 0.9% saline, was left to circulate for 10 min, and then the mouse chest was surgically opened and concurrently perfused via left (systemic circulation) and right (pulmonary circulation) ventricles with PBS for 15 min. Lungs were isolated, dry weighed, and homogenized in 200 μl of 50% TCA (dissolved in distillated water). The homogenate was frozen and kept at − 20 °C for EB concentration measurement. Subsequent to thawing, homogenates were centrifuged (at 10625 × g for 12 min at 4 °C), and the supernatant was collected and diluted with 1:3 volumes of 95% ethanol prior to photospectrometric (Synergy 4; Bio-Tek) determination of EB concentration (fluorescence: excitation at 590 nm, emission at 645 nm, absorbance at 620 nm). Results were normalized to the tissue weight.

### Western blot analysis

Lungs were perfused with PBS, excised, rinsed in saline, dried with tissue paper, weighed, cut into small pieces, and snap-frozen in liquid nitrogen; the samples were stored at − 80 °C. For western blot analysis, whole lungs were homogenized and whole lung lysates were used. Lungs were homogenized in the lysis buffer (Thermo Fisher Scientific) for protein extraction with protease and phosphatase inhibitors. Protein concentration was measured with the use of a BCA assay. Subsequently, the samples from at least six mice in each experimental group were pooled together in such a way that an equal amount of protein from each sample was dissolved in an equal volume of the lysis buffer for each mouse in the group to ensure the equal representation of each individual sample in the pooled specimen. After addition of loading buffer, samples were heated at 95 °C for 5 min and then frozen at − 80 °C. Each time, an equal amount of protein from pooled samples was loaded and run on the gel, and then transferred to a nitrocellulose membrane, blocked with 5% dry milk, and incubated with the primary antibodies directed against the following antigens: p(S1177)eNOS (ab195944; Abcam), eNOS (610,296; BD Transduction Laboratories), VEGFA (ab68334; Abcam), VEGFR2 (ab39256; Abcam), Ang-1 (ab8451; Abcam), Ang-2 (PA5-27297; Thermo Fisher Scientific), VCAM-1 (CBL1300; Merck), Slit2 (ab134166; Abcam), ROBO4 (orb101060; Biorbyt), ROBO1 (ab85312; Abcam), TGF-β1 (ab155264; Abcam), VE-CAD (sc-6458; Santa Cruz Biotechnology), CD31 (NBP1-71663H; Novus Biologicals), vWF (ab9378; Abcam), MMP-2 (ab19167; Abcam), MMP-9 (ab19016; Abcam), and MMP-14 (sab4501901; Sigma Aldrich). The appropriate horseradish peroxidase (HRP)-conjugated secondary antibodies were from Santa Cruz Biotechnology (sc-2020, sc-2004, and sc-2005). Equal protein loading was confirmed after transfer onto membranes, as measured by a stain-free technique provided by Bio-Rad [30]. Densitometric assessment of band intensity was performed using ImageJ. The results are presented as the fold change of control corresponding to healthy mice. Total protein was used as a loading control.

### Measurement of platelet basal activity and ADP-induced reactivity

Blood samples were collected into a syringe containing 3.8% citrate (blood/citrate at 10:1 (v/v)) from the right heart ventricle. A blood count was performed using the animal blood counter Vet abc (Horiba Medical, France). The samples designated for flow cytometric measurements were diluted with saline and washed with Tyrode buffer. Each sample was double stained with four antibodies that included platelet-specific antigen GpIIbIIIa (CD41/61), either FITC or PE conjugated, for platelet identification and one of four platelet activation markers—PE-conjugated active form of GPIIb/IIIa and P-selectin antibodies, FITC-conjugated fibrinogen, or von Willebrand (vWF) factor—representing platelet binding capacity. Platelets were identified based on their forward-scatter and side-scatter characteristics and were gated on the basis of the expression of platelet-specific antigen CD41/61 (see Additional file 1). Isotype control antibodies, either FITC or PE conjugated, were used to assess nonspecific binding for each individual sample. Basal and ADP-induced (20 μM) activation of circulating platelets was assessed on the basis of the measured expression/binding level of surface membrane antigens expressed as a percentage of all platelets above the isotype control fluorescent signal and the median fluorescence intensity (MFI). Flow cytometric analyses of platelet activation were performed using flow cytometry software (LSRII and FACS/Diva version 6.0, respectively; Becton Dickinson, Oxford, UK). Measurements were made on a logarithmic scale and at least 10,000 events were collected for each sample. Appropriate color compensation was determined in samples singly stained with either FITC-conjugated anti-CD41/61 or PE-conjugated anti-CD41/61.

### Statistical analysis

Data were presented as mean ± SD (box) with outliers or median of the data and interquartile range (IQR) (box, from lower (25%) to upper (75%) quartile) with outliers depending on normality of the data distribution that was tested with the Shapiro–Wilk normality test, homogeneity of variances that was tested with Barlett’s test, and the variable scale. Statistical significance was assessed with a one-way ANOVA or Kruskall–Wallis test followed by a post-hoc Tukey’s or Dunn’s multiple comparison test, respectively. Some variables nonconforming with the normal distribution and/or variance homogeneity were Box–Cox transformed and analyzed with parametric tests, otherwise they were analyzed with nonparametric inference tests. Only *P* < 0.05 was considered significant.

## Results

### Development of pulmonary metastasis and systemic inflammation in the orthotopic murine 4T1 breast cancer model

**Fig. 1:**
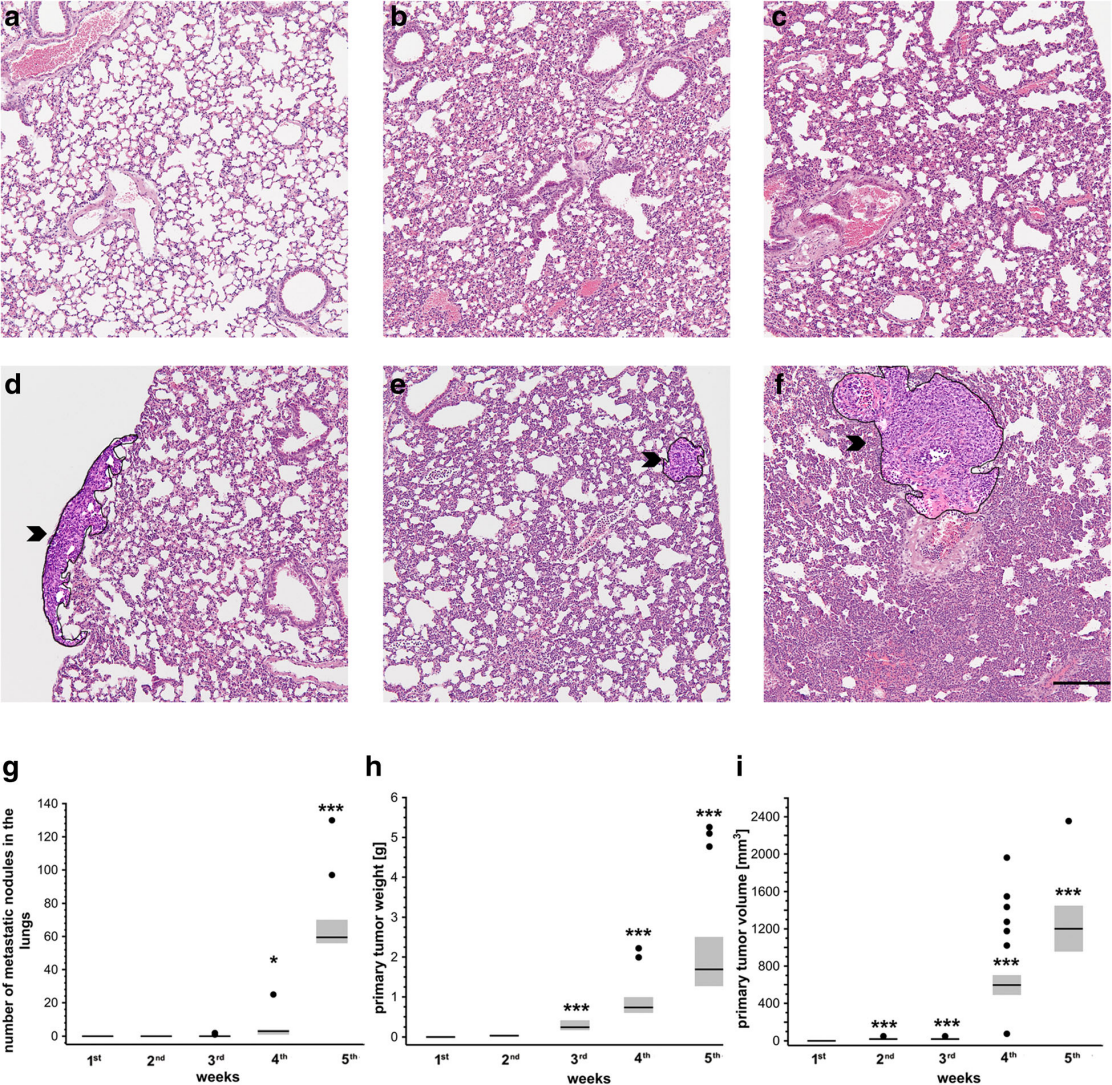
Tumor growth and development of pulmonary metastasis in 4T1 breast cancer progression in mice. **a** Lung cross-section of healthy control mouse. **b–f** Mice injected orthotopically with 4T1 breast cancer cells (see Methods)*.* Designated groups of animals sacrificed every 7 days to assess number of pulmonary metastases in 1st (**b**), 2nd (**c**), 3rd (**d**), 4th (**e**), and 5th (**f**) week after 4T1 breast cancer inoculation (black arrowheads point to metastatic nodules in lungs) on lung cross-sections stained with H&E. Scale bar represents 200 μm. **g** Number of pulmonary metastases in mice from 1st to 5th week after 4T1 cancer cell inoculation; *n* = 10 for 1st –4th week and *n* = 14 for 5th week. **h** Weight of primary tumor in consecutive weeks after 4T1 cancer cell inoculation; *n* = 30 for 1st –4th week and *n* = 68 for 5th week. (**i**) Primary tumor volume; *n* = 30 for 1st –4th week and *n* = 68 for 5th week. (g–i) Data presented as median and IQR. Black circle indicates outlier. Depending on variable scale, normality of distribution, and variance homogeneity, data analyzed with Kruskal–Wallis test followed by Dunn’s multiple comparison test. Statistical significance vs mice in 1st week after 4T1 cancer cell inoculation at *P < 0.05 and ***P < 0.001

**Fig. 2:**
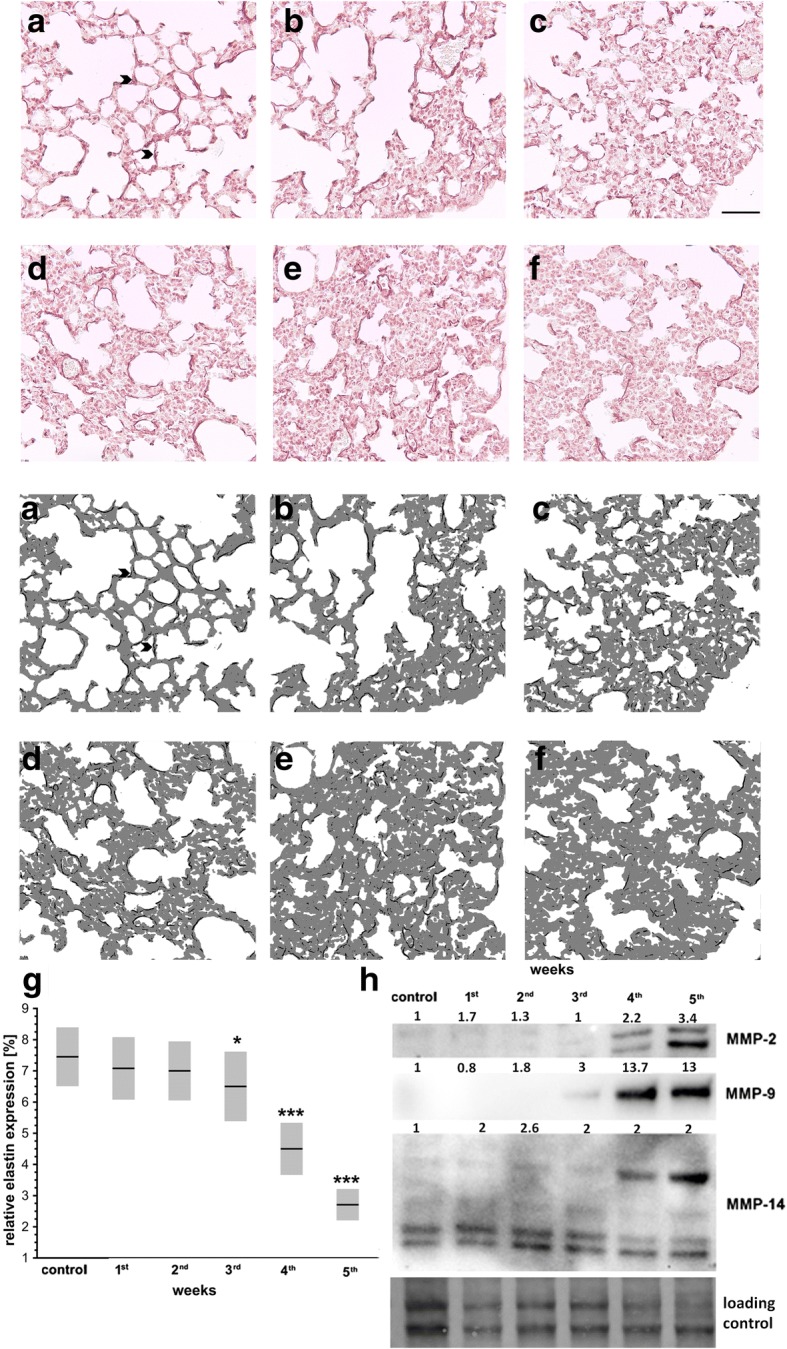
Changes in pulmonary elastin and metalloproteinase expression in 4T1 breast cancer progression in mice. Paraffin-embedded lungs cut into slices to visualize elastin (see Methods). **a–f** above Representative image of (**a**) healthy control and 1st (**b**), 2nd (**c**), 3rd (**d**), 4th (**e**), and 5th (**f**) week after 4T1 cancer cell inoculation. Scale bar represents 50 μm. **a**–**f** below Corresponding segmentation. **g** Differences in relative elastin expression (dark gray pixels corresponding to elastin vs light gray pictures corresponding to lung tissue), shown as mean ± SD. Black circle indicates outlier; *n* = 20 for healthy control mice and mice in 1st, 2nd, and 4th week of disease; *n* = 21 for mice in 3rd week of disease; *n* = 19 for mice in 5th week of disease. Data subjected to Box–Cox transformation and analyzed by one-way AVOVA followed by Tukey’s multiple comparison test due to normality of distribution and homogeneity of variances. Statistical difference vs healthy control mice at *P < 0.05 and ***P < 0.001. **h** MMP-2, MMP-9, and MMP-14 expression in pooled samples (*n* = 6) (see Methods). MMP-2, higher band indicates inactive isoform while lower band indicates active isoform. MMP-14, higher band corresponds to active monomer while lower band corresponds to domains after catalytical cleavage. Results presented as fold change vs control sample corresponding to healthy mice. Total protein after transfer was used as loading control

The first pulmonary metastatic nodules were detected in the 3rd week after breast cancer cell injection (Fig. 1d, g), while the primary tumor became detectable in the 2nd week after cancer cell inoculation (Fig. 1i). Then, both the number of pulmonary metastases and the primary tumor weight and volume increased progressively (Fig. 1). The weight of the lungs was significantly increased only in the 5th week compared to control healthy mice (Table 1), since only at that time was the presence of large metastastic foci in the lungs detected (Fig. 1f, g). The appearance of the first metastatic nodules in the lungs in the 3rd week after breast cancer cell injection correlated with the onset of systemic leukocytosis and increased spleen weight (Table 1), indicating the onset of systemic inflammation. Pulmonary metastasis was also associated with progressive degradation of elastin fibers (Fig. 2a–g) and an increase in the expression of metalloproteinase 2, 9, and 14 (MMP-2, MMP-9, MMP-14) in the lungs 3 and 4 weeks after breast cancer cell injection, respectively (Fig. 2h), being compatible with tissue remodeling accompanying the advanced stage of metastasis progression.

**Table 1:** Lung/spleen weight and blood count in the orthotopic 4T1 breast cancer model in mice

Parameter	Week after 4T1 cancer cell inoculation					
	Control	1st	2nd	3rd	4th	5th
Lung weight (g)	0.76; 0.70–0.80 (*n* = 30)	0.77; 0.70–0.81 (*n* = 29)	0.76; 0.72–0.84 (*n* = 30)	0.81; 0.76–0.85 (*n* = 29)	0.0.82; 0.77–0.91 (*n* = 30)	1.7; 1.3–2.22*** (n = 49)
WBC (K/μl)	4.20; 3.41–5.22 (*n* = 35)	4.70; 4.04–5.28 (*n* = 21)	5.55; 4.94–6.21 (*n* = 23)	17.02; 11.85–24.78*** (*n* = 23)	54.62; 96.18–177.10*** (*n* = 20)	257.30; 148.20–317.10*** (*n* = 36)
GRA (K/μl)	1.00; 0.80–1.20 (*n* = 35)	1.25; 1.10–1.40 (*n* = 21)	1.85; 1.35–2.40* (*n* = 23)	11.30; 6.95–16.10*** (*n* = 23)	79.00; 42.48–132.80*** (*n* = 20)	159.80; 89.25–220.90*** (*n* = 36)
LYM (K/μl)	3.10; 2.50–3.85 (*n* = 35)	3.25; 2.75–3.87 (*n* = 21)	3.40; 2.80–3.90 (*n* = 23)	5.65; 4.15–7.00*** (*n* = 23)	18.50; 9.75–24.13*** (*n* = 20)	46.90; 25.00–95.00*** (*n* = 35)
Spleen weight (g)	0.10; 0.09–0.11 (*n* = 20)	0.10; 0.09–0.11 (*n* = 30)	0.12; 0.11–0.13 (*n* = 30)	0.24; 0.20–0.32** (*n* = 30)	0.54; 0.43–0.68*** (*n* = 30)	0.89; 0.79–0.99*** (*n* = 70)

Data presented as median; interquartile range. Blood count performed to assess development of systemic inflammation whereas spleen weight recorded as an indirect marker of systemic inflammation [48]. Based on normality of distribution and variance homogeneity, data were analyzed with Kruskal–Wallis test followed by Dunn’s multiple comparison test. Statistical significance vs healthy control mice at *P < 0.05, **P < 0.01 and ***P < 0.001

*WBC* white blood cells, *GRA* granulocytes, *LYM* lymphocytes

### Progressive impairment of NO production in lungs in the orthotopic murine 4T1 breast cancer model

**Fig. 3:**
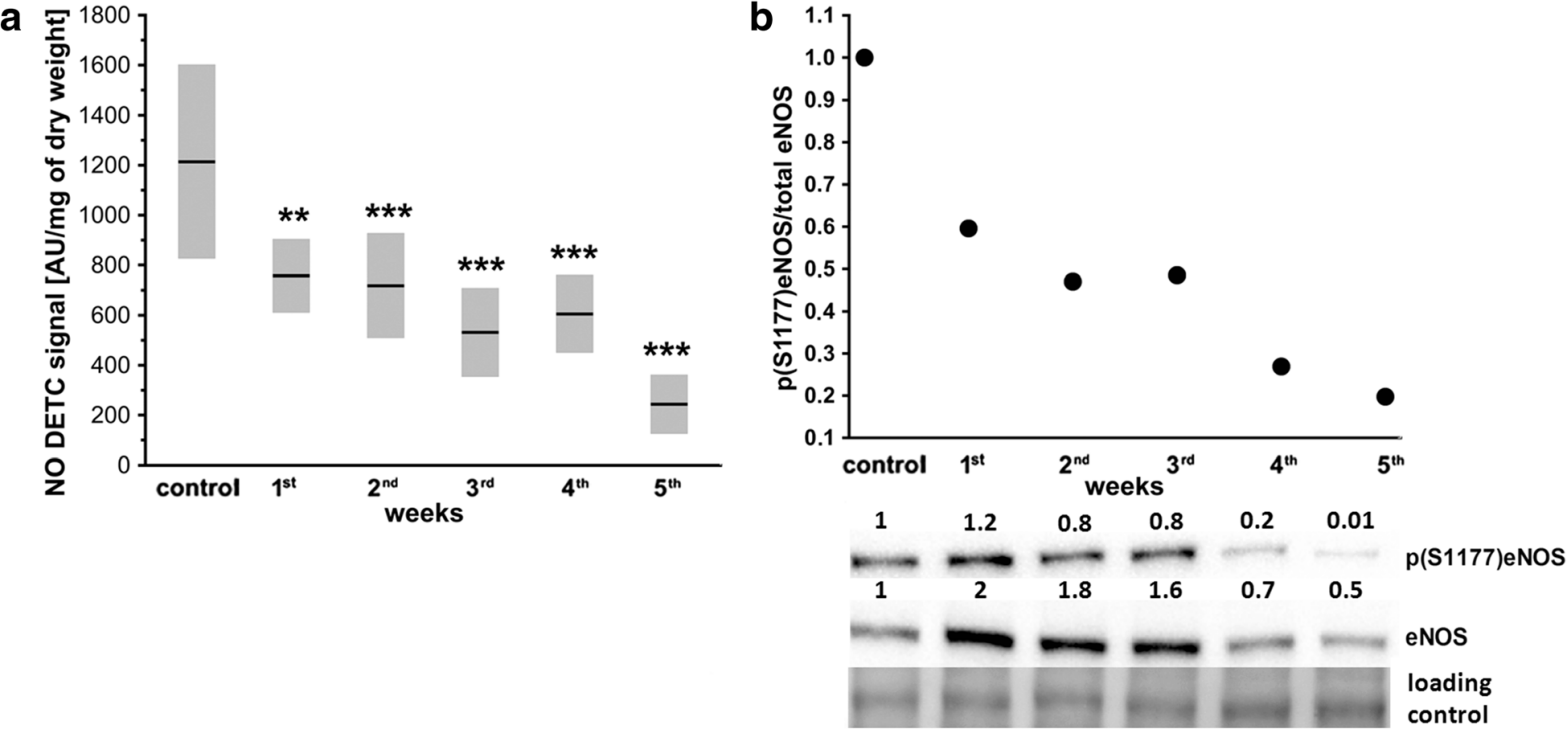
Pulmonary NO production and eNOS phosphorylation in orthotopic 4T1 breast cancer model in mice. **a** NO production in lungs, presented as mean ± SD. Data were Box–Cox transformed and analyzed with one-way ANOVA followed by Tukey’s multiple comparison test; *n* = 10 for control and 1st–4th week of disease; *n* = 19 for 5th week of disease. Black circle indicates outlier. Perfused lungs were excised from euthanized animals and NO production measured (see Methods). Statistical significance vs healthy control mice at **P < 0.01 and ***P < 0.001. **b** Densitometric data presenting eNOS and p(S1177)eNOS levels during progression of breast cancer used to calculate relative eNOS phosphorylation at serine 1177 (black circle) expressed as fraction of total eNOS level in lungs of control healthy mice for which an arbitrary value of 1 was ascribed. Western blot image presents fold change vs control sample corresponding to healthy mice. Total protein after transfer was used as loading control. Western blot image shows p(S1177)eNOS and eNOS levels in pooled samples in all experimental groups obtained by pooling lung homogenates from six mice in each experimental group (see Methods). AU arbitrary units, eNOS endothelial nitric oxide synthase, NO nitric oxide

Local NO production in the lungs was impaired already in the 1st week after 4T1 cell injection (Fig. 3a) and remained significantly compromised thereafter in 4T1 breast cancer-bearing mice. The progressive fall in NO production in mice injected with 4T1 cells corresponded with progressive decrease in eNOS phosphorylation of S1177 observed throughout the progression of the disease after 4T1 cancer cell inoculation (Fig. 3b).

### Expression of endothelial–mesenchymal transition markers in pulmonary endothelium in the orthotopic murine 4T1 breast cancer model

**Fig. 4:**
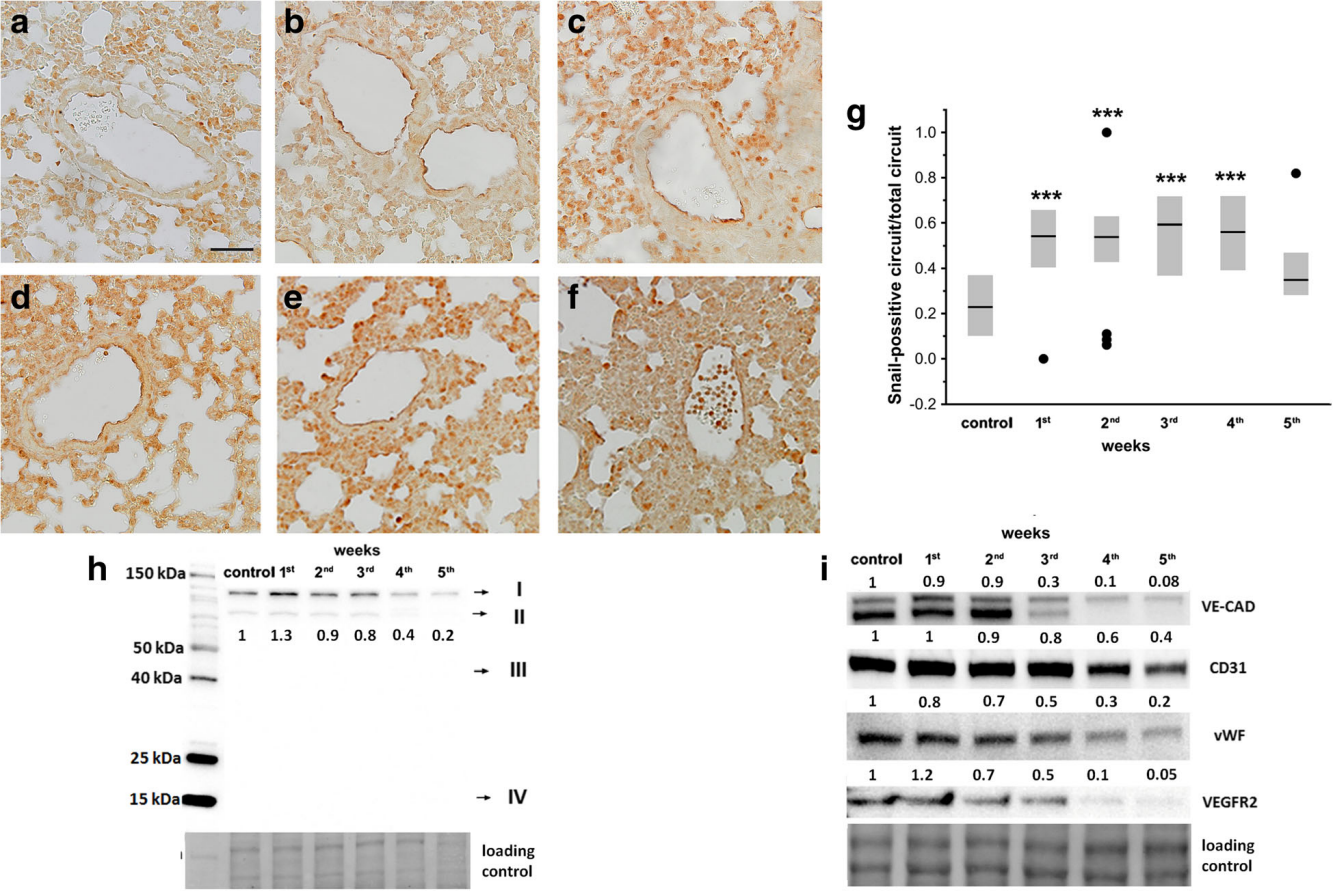
Snail and EndMT-related protein expression in lungs in orthotopic murine 4T1 breast cancer model. **a–g** Expression of Snail (see Methods). Representative micrographs of arteries from control (**a**), 1st (**b**), 2nd (**c**), 3rd (**d**), 4th (**e**), and 5th (**f**) week after 4T1 cell inoculation. Scale bar represents 50 μm. **g** Quantitative expression of Snail in endothelial layer of small arteries of each group, shown as median and IQR. Black circle indicates outlier. Data analyzed with Kruskal–Wallis test followed by Dunn’s multiple comparison test; *n* = 61, *n* = 60, *n* = 58, *n* = 60, *n* = 60, and *n* = 59 for control, 1st, 2nd, 3rd, 4th and 5th week. Statistical significance vs healthy control group at ***P < 0.001. **h, i** TGF-β1 (I, TGF-β1 and TGF-β2 heterodimers; II, TGF-β1 homodimers; III, full-length inactive TGF-β1; IV, mature TGF-β1) (**h**) and VE-CAD (vascular endothelium cadherin), CD31 (cluster of differentiation 31), vWF (von Willebrand factor), and VEGFR2 (vascular endothelial growth factor receptor 2) (**i**) levels determined by western blot analysis in pooled (*n* = 6) samples in control and 4T1 breast cancer-bearing mice from the 1st to 5th week after 4T1 cancer cell inoculation (see Methods). Results presented as fold change vs control sample corresponding to healthy mice. Total protein after transfer was used as loading control

Expression of Snail in the lungs of mice orthotopically injected with 4T1 breast cancer cells, compared with healthy control mice, was higher in the endothelial layer of small arteries as soon as 1 week after cancer cell injection and stayed elevated throughout the entire progression of the disease, except for the terminal time point 5 weeks after 4T1 breast cancer cell inoculation (Fig. 4a–g). TGF-β1 expression in the lungs was slightly increased only in the 1st week after 4T1 breast cancer cell inoculation (Fig. 4h). The phenotypic change of pulmonary endothelium compatible with endothelial–mesenchymal transition, evidenced by downregulation of endothelium-specific proteins such as VE-CAD, CD31, vWF, or VEGFR2, seemed to be evident 3 weeks after cancer cell inoculation (Fig. 4i), concomitant with the early phase of metastasis (Fig. 1g).

### Changes in pulmonary endothelial barrier function in the orthotopic murine 4T1 breast cancer model

**Fig. 5:**
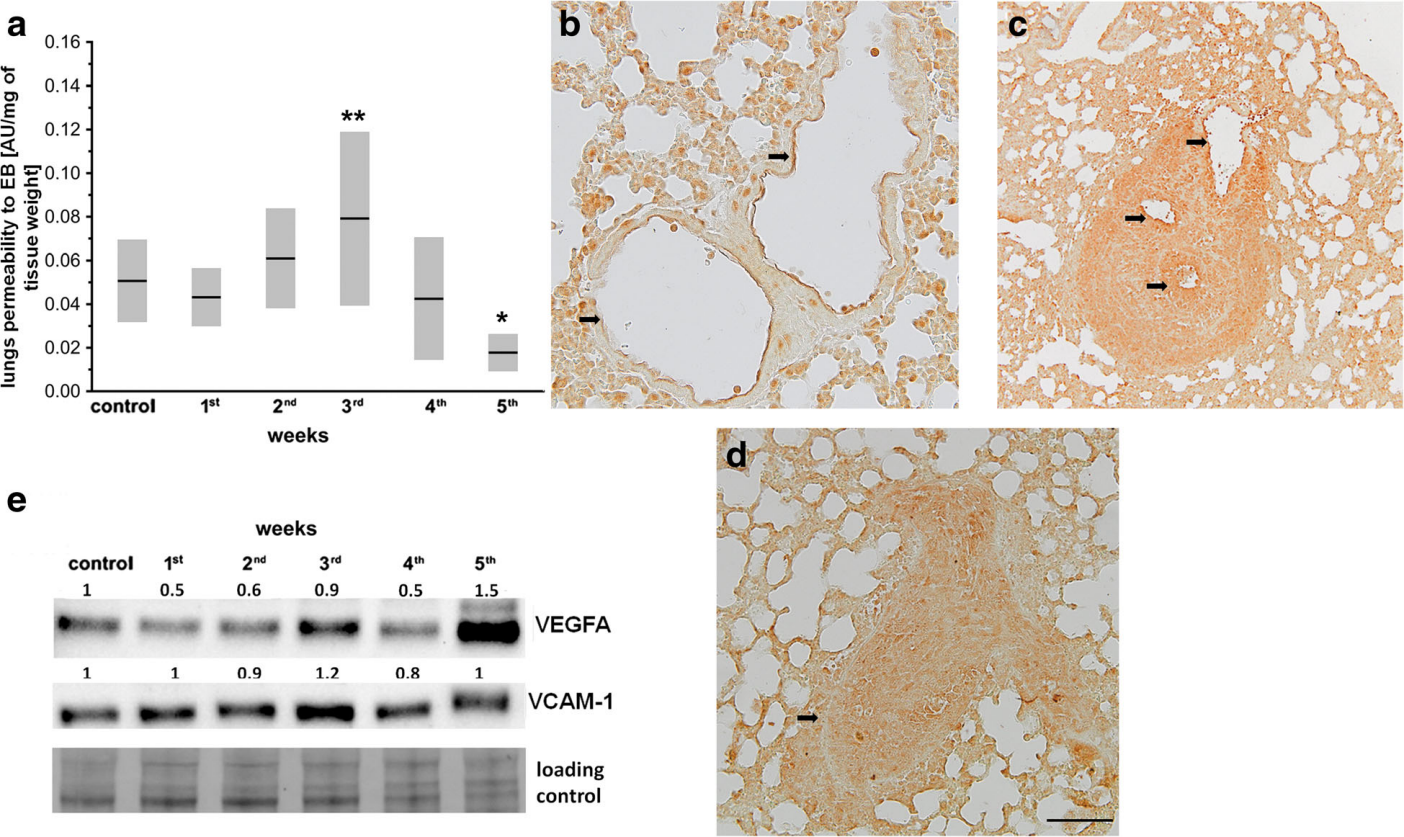
Pulmonary endothelium permeability in orthotopic murine 4T1 breast cancer model. **a** Evans blue (EB) deposition in lungs (see Methods), presented as mean ± SD. Black circle indicates outlier. Data analyzed with one-way ANOVA followed by Tukey’s multiple comparison test based on normality of distribution and homogeneity of variances; *n* = 20 for control and 1st and 3rd week; *n* = 18 for 2nd and 4th week; *n* = 10 for 5th week. Statistical significance vs healthy control group at *P < 0.05 and P** < 0.01. **b–d** Lung slices stained to visualize transcription factor Snail (see Methods), which is highly expressed in secondary nodules composed of metastatic 4T1 breast cancer cells. Black arrows point to pulmonary blood vessels of mice in 3rd (**b**), 4th (**c**), and 5th (**d**) week after 4T1 cancer cell inoculation to visualize that in 4th and 5th weeks some pulmonary vessels were occluded by metastatic cancer cells proliferating in their lumen. Scale bar represents 50 μm. **e** VEGFA and VCAM-1 expression determined by western blot analysis in pooled samples (*n* = 6) corresponding to healthy control and 4T1 breast cancer-bearing mice in 1st–5th week after 4T1 cancer cell inoculation (see Methods). Results presented as fold change vs control sample. Total protein after transfer was used as loading control. AU arbitrary units, VCAM vascular cell adhesion molecule, VEGFA vascular endothelial growth factor A

Increased permeability of pulmonary endothelium expressed as increased deposition of Evans blue (EB) in the lungs of 4T1 breast cancer-bearing mice was found only in the 3rd week after cancer cell inoculation (Fig. 5a), indicating an evident increase in endothelial permeability at the early phase of metastasis. Then, EB leakage from the circulation into the lungs started to decrease and, finally, it was lower than in healthy control mice in the 5th week after cancer cell inoculation. Decreasing EB penetration into the lungs of mice in the 4th and 5th weeks of the disease appeared to be due to the occlusion of pulmonary vessels by cancer cells proliferating in their lumen (Fig. 5b–d), rather than due to changes in pulmonary endothelial permeability itself. Increased permeability of the pulmonary endothelium in the 3rd week after cancer cell inoculation correlated with a transient increase in VEGFA and VCAM-1 expression in the lungs (Fig. 5e).

### Progressive downregulation of Slit2–ROBO4–ROBO1 pathway in the orthotopic murine 4T1 breast cancer model

**Fig. 6:**
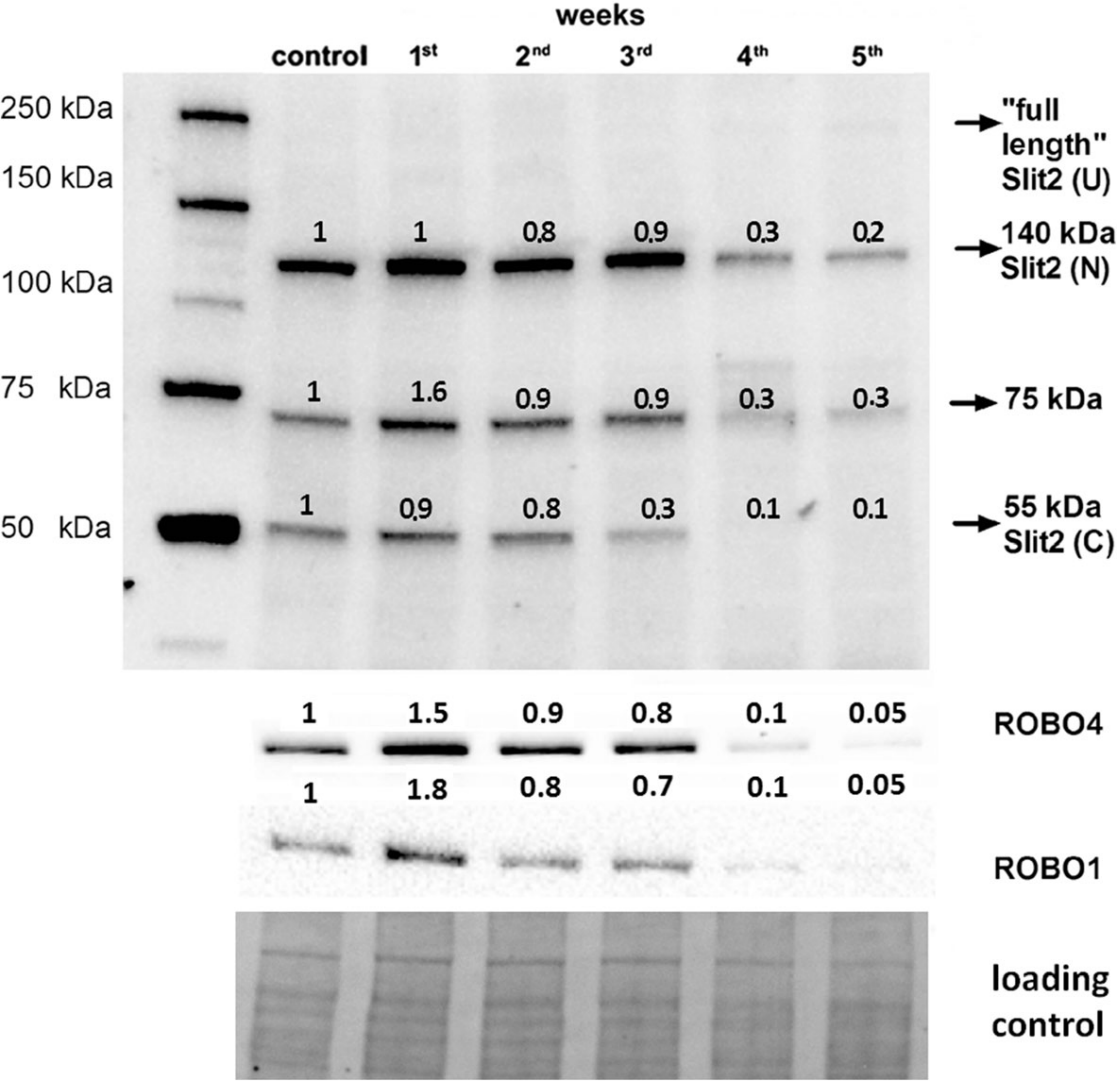
Slit2–ROBO4–ROBO1-dependent signaling in orthotopic murine 4T1 breast cancer model. Slit2 (axon guidance molecule), ROBO4 and ROBO1 (roundabout 1 and 2) expression determined by western blot analysis in pooled samples (*n* = 6) corresponding to healthy control and 4T1 breast cancer-bearing mice from 1st to 5th week after 4T1 cancer cell inoculation (see Methods). Full-length Slit2 ~ 200 kDa (U) is proteolytically cleaved, giving rise to an N-terminal fragment ~ 140 kDa (N) and a C-terminal fragment ~ 55 kDa (C). Only full-length and N-terminal peptides can bind ROBO proteins. Therefore, representative picture of entire membrane probed with primary anti-Slit2 antibody presented; we are unsure about identity of the ~ 75 kDa band detected with anti-Slit2 antibody. Results presented as fold change vs control sample corresponding to healthy mice. Total protein after transfer was used as loading control. ROBO roundabout family of receptors, Slit axon guidance molecule

Slit2 maintains endothelium integrity via interaction of its full-length or N-terminal part with endothelium-specific ROBO4 via ROBO1 [19, 31, 32]. Full-length Slit2 was undetectable in lungs homogenates of both control and 4T1 breast cancer-bearing mice, while its N-terminal fragment was found both in healthy control and breast-cancer bearing mice (Fig. 6). Upregulation of both receptors ROBO4 and ROBO1 was detected only in the 1st week after 4T1 cell injection when the level of N-terminal Slit2 binding ROBO receptors was still preserved. Starting from the 2nd week after cancer cell inoculation, the Slit2–ROBO4–ROBO1 protective signaling pathway was gradually downregulated in the lungs of 4T1 breast cancer-bearing mice.

### Changes in basal platelet activation and ADP-induced reactivity in the orthotopic murine 4T1 breast cancer model

**Fig. 7:**
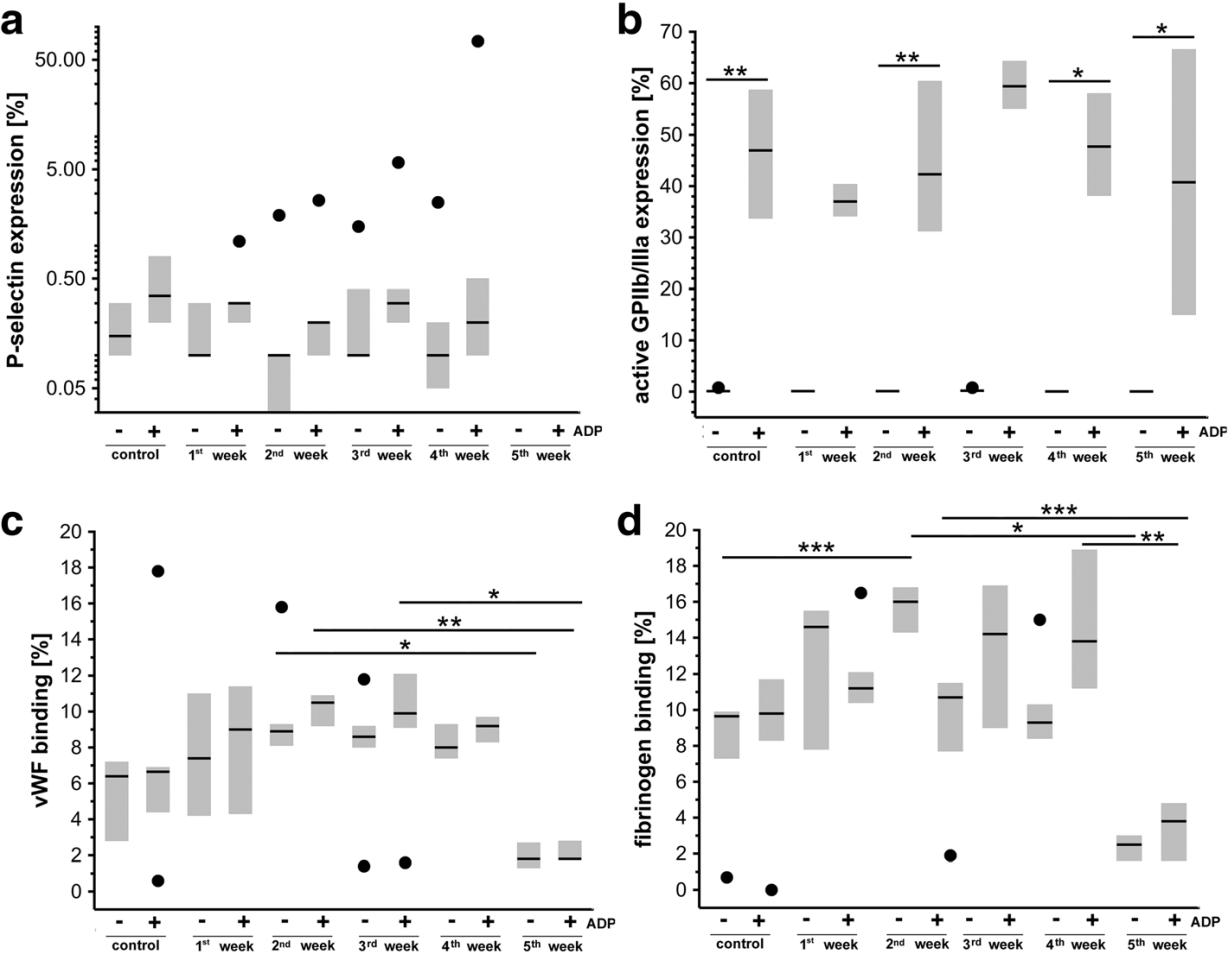
Platelet basal activity and ADP-induced reactivity in orthotopic murine 4T1 breast cancer model. Platelet basal activation and their ADP-induced reactivity assessed as percentage of platelet surface expression of P-selectin (**a**), active form of receptor GPIIb/IIIa (**b**), von Willebrand factor (vWF) binding (**c**), and fibrinogen binding (**d**) (see Methods), presented as median and IQR. Black circle indicates outlier. Data analyzed with Kruskal–Wallis test followed by Dunn’s multiple comparison test since they either did not display normal distribution and/or their variances were heterogeneous. **a** *n* = 10, *n* = 7, *n* = 9, *n* = 7, *n* = 8, and *n* = 12 for healthy control, 1st, 2nd, 3rd, 4th, and 5th week, respectively; and *n* = 9, *n* = 7, *n* = 8, *n* = 6, *n* = 6, and *n* = 12 for healthy control, 1st, 2nd, 3rd, 4th, and 5th week after ADP stimulation. **b** *n* = 10, *n* = 7, *n* = 9, *n* = 7, *n* = 8, and *n* = 13 for healthy control, 1st, 2nd, 3rd, 4th, and 5th week, respectively; and *n* = 10, *n* = 7, *n* = 9, *n* = 7, *n* = 7, and *n* = 13 for healthy control, 1st, 2nd, 3rd, 4th, and 5th week after ADP stimulation. **c** *n* = 10, *n* = 7, *n* = 8, *n* = 7, *n* = 8, and *n* = 18 for healthy control, 1st, 2nd, 3rd, 4th, and 5th week, respectively; and *n* = 10, *n* = 7, *n* = 9, *n* = 7, *n* = 7, and *n* = 19 for healthy control, 1st, 2nd, 3rd, 4th, and 5th week after ADP stimulation. **d** *n* = 10, *n* = 7, *n* = 8, *n* = 7, *n* = 8, and *n* = 20 for healthy control, 1st, 2nd, 3rd, 4th, and 5th week, respectively; and *n* = 9, *n* = 7, *n* = 9, *n* = 7, *n* = 8, and *n* = 20 for healthy control, 1st, 2nd, 3rd, 4th, and 5th week after ADP stimulation. Statistical significance at level of *P < 0.05, **P < 0.01, and ***P < 0.001

**Fig. 8:**
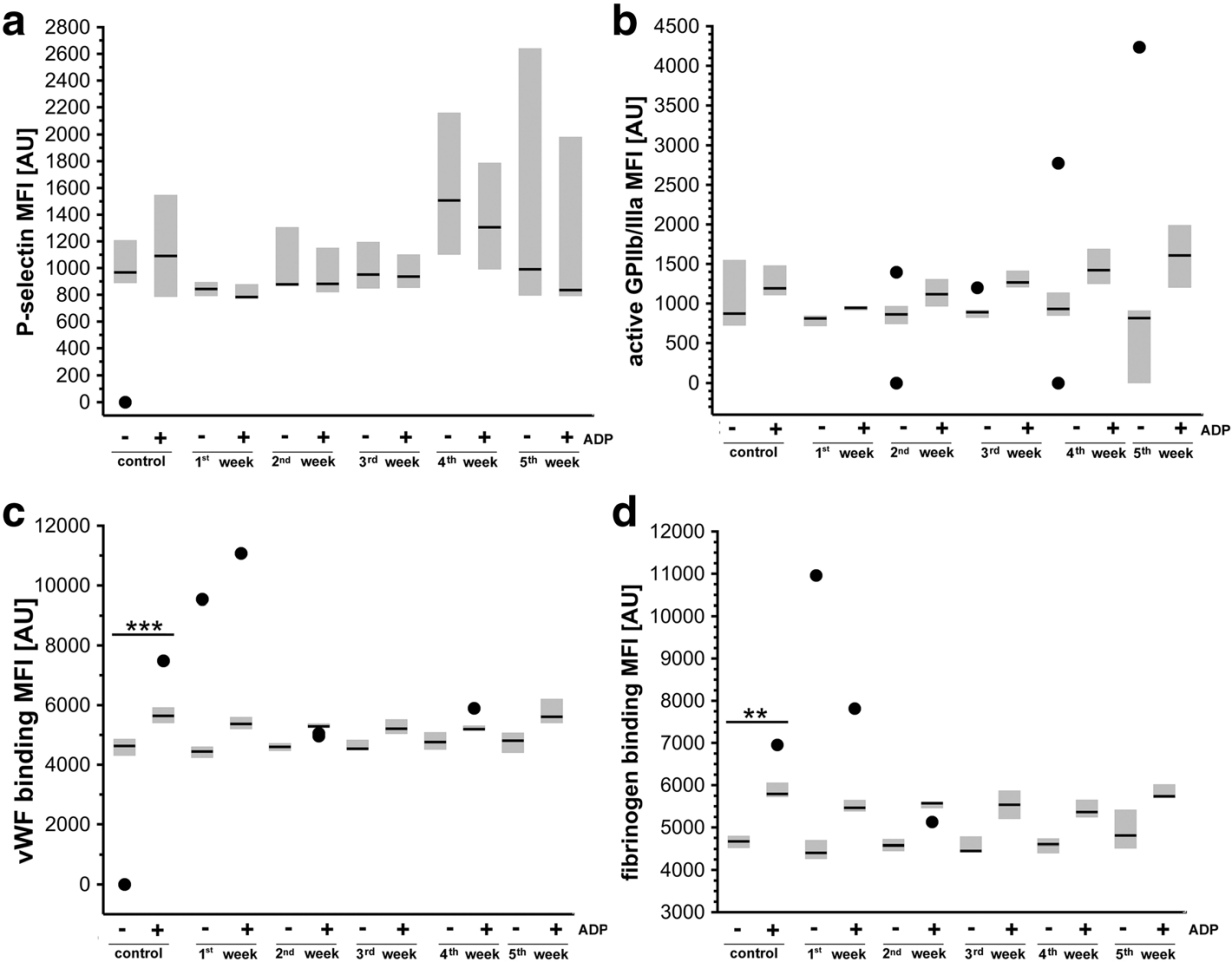
Basal and ADP-stimulated MFI of platelet antigenes in orthotopic murine 4T1 breast cancer model. Median expression of P-selectin (**a**), active form of GPIIb/IIIa (**b**), bound von Willebrand factor (vWF) (**c**), and fibrinogen (**d**) shown as median fluorescence intensity (MFI). Data presented as median and IQR. Black circle indicates outlier. Data analyzed with Kruskal–Wallis test followed by Dunn’s multiple comparison test since they either did not display normal distribution and/or their variances were heterogeneous. **a** *n* = 10, *n* = 7, *n* = 9, *n* = 7, *n* = 8, and *n* = 12 for healthy control, 1st, 2nd, 3rd, 4th, and 5th week, respectively; and *n* = 9, *n* = 7, *n* = 8, *n* = 6, *n* = 6, and *n* = 12 for healthy control, 1st, 2nd, 3rd, 4th, and 5th week after ADP stimulation. **b** *n* = 10, *n* = 7, *n* = 9, *n* = 7, *n* = 8, and *n* = 13 for healthy control, 1st, 2nd, 3rd, 4th, and 5th week, respectively; and *n* = 10, *n* = 7, *n* = 9, *n* = 7, *n* = 7, and *n* = 13 for healthy control, 1st, 2nd, 3rd, 4th, and 5th week after ADP stimulation. **c** *n* = 10, *n* = 7, *n* = 8, *n* = 7, *n* = 8, and *n* = 18 for healthy control, 1st, 2nd, 3rd, 4th, and 5th week, respectively; and *n* = 10, *n* = 7, *n* = 9, *n* = 7, *n* = 7, and *n* = 19 for healthy control, 1st, 2nd, 3rd, 4th, and 5th week after ADP stimulation. **d** *n* = 10, *n* = 7, *n* = 8, *n* = 7, *n* = 8, and *n* = 20 for healthy control, 1st, 2nd, 3rd, 4th, and 5th week, respectively; and *n* = 9, *n* = 7, *n* = 9, *n* = 7, *n* = 8, and *n* = 20 for healthy control, 1st, 2nd, 3rd, 4th, and 5th week after ADP stimulation. Statistical significance at level of **P < 0.01 and ***P < 0.001. AU arbitrary units

Basal platelet activation and ADP-induced reactivity were both assessed as the percentage of platelets expressing P-selectin, active form of GPIIb/IIIa, vWF, and fibrinogen bound to platelet surface in their entire population (Fig. 7) as well as median expression of these antigens on the platelet surface (Fig. 8). The signs for activation of platelets in the early phase of metastasis and loss of reactivity to stimuli afterward were detected. The percentage of platelets expressing P-selectin and active form of GPIIb/IIIa peptide in basal condition or upon ADP stimulation were not different between healthy control and 4T1 breast cancer-bearing mice (Fig. 7a, b). However, fibrinogen binding in basal conditions was increased in 4T1 breast cancer-bearing mice as compared with control mice 1–3 weeks after 4T1 cancer cell inoculation, reaching significance 2 weeks after cancer cell inoculation. Similarly, vWF binding to platelets was also increased 1–3 weeks after cancer cell inoculation (not significantly). In contrast, 5 weeks after 4T1 breast cancer cell inoculation, the capacity of platelets to bind fibrinogen and vWF was significantly diminished in basal conditions as well as after ADP stimulation (Fig. 7c, d). As shown in Fig. 8, median expression of P-selectin, active form GPIIb/IIIa, vWF, and bound fibrinogen on the unstimulated platelet surface was not altered in 4T1 breast cancer-bearing mice as compared with controls. However, after ADP stimulation, platelet surface expression of vWF and fibrinogen but not P-selectin and active form of GPIIb/IIIa was increased to a lesser extent in 4T1 breast cancer-bearing mice as compared with healthy controls (Fig. 8c, d).

## Discussion

This study comprehensively characterized step-by-step progression of pulmonary endothelial dysfunction during 4T1 breast cancer growth and metastasis to establish at which stage endothelial impairment and mesenchymal transformation of endothelial cells (EndMT) occurs in metastatic organs in relation to the formation of metastasis. We found that NO production in pulmonary endothelium was impaired already at the premetastatic phase of the disease concomitantly with decreased eNOS phosphorylation in the lungs (Fig. 3). However, even though the expression of Snail transcription factors in the pulmonary endothelium (Fig. 4a–g) and TGF-β1 in the lungs (Fig. 4h) (both known to be main drivers of EndMT) were increased in relation to healthy controls already in the premetastatic stage (1st week), the onset of functional phenotypic switch of pulmonary endothelium known as EndMT seemed to take place no earlier than the 3rd week after breast cancer cell inoculation (Fig. 4i). At that stage, pulmonary endothelium became activated, as evidenced by higher VCAM-1 expression (Fig. 5e), and endothelial barrier integrity was lost, as visualized by increased deposition of EB in the lungs (Fig. 5a). Development of EndMT also seemed to coincide with progressive decrease in pulmonary elastin levels (Fig. 2a–g) and increased expression of proteolytic enzymes like metalloproteinase 2, 9, and 14 (MMP-2, MMP-9, MMP-14) in the lungs (Fig. 2h), indicating metastatic tissue remodeling at this stage. To summarize, our results identified decreased eNOS activity and phosphorylation resulting in an NO-deficiency state as an early event in breast cancer pulmonary metastasis, that occurs prior to the decrease in expression of endothelium-specific markers that indicates functional phenotypic switch of pulmonary endothelium toward a mesenchymal phenotype (EndMT), coinciding with development of the first metastatic nodules in the lungs and metastatic tissue remodeling.

Recently, it has been postulated that endothelial cells can actively augment metastatic extravasation through the shift in their phenotype known as endothelial–mesenchymal transition [26]. Moreover, in the case of the lungs, transformed endothelial cells can represent a significant source of fibroblasts [33] that can enrich the population of cancer-associated fibroblasts (CAFs) that constitute key components of tumor stroma [26]. Therefore, hampering or preventing EndMT could directly affect settlement of cancer cells in the lungs. In the present work, however, we demonstrated that the phenotypic switch of pulmonary endothelium toward mesenchymal cells (EndMT, evidenced by downregulation of VE-CAD, CD31, vWF, or VEGFR2 [34]) is a relatively late response in murine 4T1 metastatic breast cancer. It coincides with increased endothelial permeability and early metastasis but is preceded by a clear-cut NO-deficiency state, that was detected as early as 1 week after 4T1 cancer cell inoculation in the premetastatic stage, even before the primary tumor was detectable. These findings suggest that early endothelial dysfunction in the lungs, indicated by NO deficiency rather than EndMT, might represent a primary regulatory target to prevent early pulmonary metastasis. Indeed, NO was shown to inhibit heteroadhesion of cancer cells to endothelial cells [5]. Moreover, decreased NO levels in the circulation have been shown to promote EndMT [35], while eNOS stimulation had the opposite effect [36]. However, the role of NO bioavailability in regulation of progression of various cancers is complex and its antimetastatic effects seem to depend on multiple factors (i.e., disease stage [37]). Indeed, while Buczek et al. [38] showed that both local as well as systemic NO deficiency was present in the premetastatic stage of 4T1 breast cancer, increased systemic NO bioavailability at the advanced stage was associated with increased mortality of 4T1 breast cancer-bearing mice [39].

Interestingly, in spite of early NO deficiency (Fig. 3), the onset of functional EndMT in the lungs of 4T1 breast cancer-bearing mice seemed to be delayed until the 3rd week, in spite of the increased expression of Snail transcription factors in the pulmonary endothelium (Fig. 4a–g) and TGF-β1 in the lungs (Fig. 4h) known to drive mesenchymal shift of endothelial cells [22]. We are tempted to speculate that the delayed onset of EndMT could result from the activation of Slit2 and its receptors’ ROBO1–ROBO4-dependent signaling in the 1st week of breast cancer progression (Fig. 6), since Slit2 was previously reported to inhibit both TGF-β [40] and Snail [41] signaling, both involved in the triggering of mesenchymal transformation of endothelial cells [20, 22–25, 34]. When expression of Slit2 receptors started to decline progressively (2nd week of disease) (Fig. 6), a similar trend was observed in the case of VE-CAD, CD31, vWF, or VEGFR2 (Fig. 4i), indicating the onset of functional EndMT [34]. However, although this new role of Slit2-dependent signaling in inhibition of EndMT in malignant disease is emerging, mechanistic confirmation is still missing. One possibility involves Slit2-dependent inhibition of Notch signaling [42] that is known to trigger EndMT [43]. Last but not least, delayed onset of EndMT in the lungs of 4T1 breast cancer-bearing mice could also have been associated with other Slit2-independent signaling pathways, such as bFGF-dependent signaling counteracting TGF-β signaling [44].

The initiation of functional EndMT in the lungs of breast cancer-bearing mice that seemed to take place at the beginning of the metastatic phase did not coincide with the rise in TGF-β1 levels in the lungs at that time (Fig. 4h) and, therefore, must have been associated with activation of TGF-β-independent mechanisms. Recently, Krenning et al. [22] reported that EndMT could also be triggered by disturbances in shear stress and activation of mechanoreceptors such as VE-CAD, CD31, or VEGFR2 on endothelial cells. The level of VEGFA in the lungs of 4T1 breast cancer-bearing mice was transiently increased in the 3rd week of the disease (Fig. 5e) and such a local rise in VEGFA has been recently associated with oscillatory turbulent flow (Lena Claesson-Welsh, personal communication, the 2nd Nov, 2016) that could activate the mechanoreceptors on endothelial cells. Last but not least, yet another possible trigger for EndMT in pulmonary metastasis and pulmonary remodeling is hypoxia. Therefore, further investigation of signaling pathways involved in triggering EndMT of pulmonary endothelium in breast cancer progression is needed, but is beyond the scope of this work.

Effective extravasation of cancer cells could also be actively promoted by platelets. Once activated, platelets express on their surface or release various tumor-promoting factors, protect circulating tumor cells (CTCs) from immune attack, or promote extravastation of CTCs/tumor angiogenesis [10]. In the present work, we found the signs for activation of platelets in the early phase of metastasis (Fig. 7c, d) and very early loss of their reactivity in response to ADP ex vivo (Fig. 8c, d), confirming a possible involvement of platelets in the early host response to circulating cancer cells [10, 45]. However, although activation of platelets initiates vascular inflammation associated with various diseases [15], their contribution to early pulmonary endothelial dysfunction and/or EndMT in breast cancer progression still needs to be verified.

## Conclusions

This study comprehensively described progression of endothelial dysfunction in the lungs, being the primary site of metastasis in Balb/C mice bearing breast cancer. The major finding was that pulmonary endothelium dysfunction, in terms of compromised NO production and decreased eNOS phosphorylation, was an early event in breast cancer progression. It preceded the decrease in expression of endothelium-specific proteins indicating onset of the phenotypic switch of pulmonary endothelium toward the mesenchymal phenotype (EndMT) and development of first metastatic nodules in the lungs. These findings suggest that early endothelial dysfunction featured by NO deficiency in the lungs rather than EndMT might represent a primary regulatory target to prevent early pulmonary metastasis. However, further studies are needed to establish whether preventing the NO deficiency in the function of pulmonary endothelium at the premetastatic stage (i.e., by locally increasing NO bioavailability) could indeed delay or inhibit pulmonary metastasis. It also remains a matter of debate whether targeting pulmonary endothelial mesenchymal transition at the metastatic stage of a malignant disease (e.g. by inhibiting mesenchymal transformation of endothelial cells as by preventing IL-1β-dependent signaling [46, 47]) could improve disease outcomes.

## Additional file

Additional file 1: Platelet population gating in LSRII using FACS/Diva version 6.0 software. Gating strategy for one of four antibodies where platelets positive for GpIIbIIIa (CD41/61) express active form of GpIIbIIIa after stimulation with ADP (20 μM). Population of platelets selected from diluted whole blood sample in two steps: firstly, based on forward-scatter (FFC) and side-scatter (SSC) characteristics; secondly, based on CD41/61 antigen positive expression. Finally, expression level of activation marker (GpIIbIIIa active form) measured in selected population of platelets. (TIF 132 kb)

## Abbreviations

Ang: Angiopoetin
CD31: Cluster differentiation 31
EndMT: Endothelial–mesenchymal transition
eNOS: Endothelial nitric oxide synthase
IQR: Interquartile range
MFI: Median fluorescence intensity
MMP: Metalloproteinase
ROBO: Roundabout family of receptors
Slit: Axon guidance molecule
TGF-β: Transforming growth factor beta
VCAM: Vascular cell adhesion molecule
VE-CAD: Vascular endothelial-cadherin
VEGFA: Vascular endothelial growth factor A
VEGFR2: Vascular endothelial growth factor receptor 2
vWF: Von Willebrand factor
