# Supplementary figures and images for: Nitric oxide deficiency and endothelial–mesenchymal transition of pulmonary endothelium in the progression of 4T1 metastatic breast cancer in mice
Source: Breast Cancer Res. 2018 Aug 3;20:86. doi: 10.1186/s13058-018-1013-z (PMC6091065; doi:10.1186/s13058-018-1013-z)

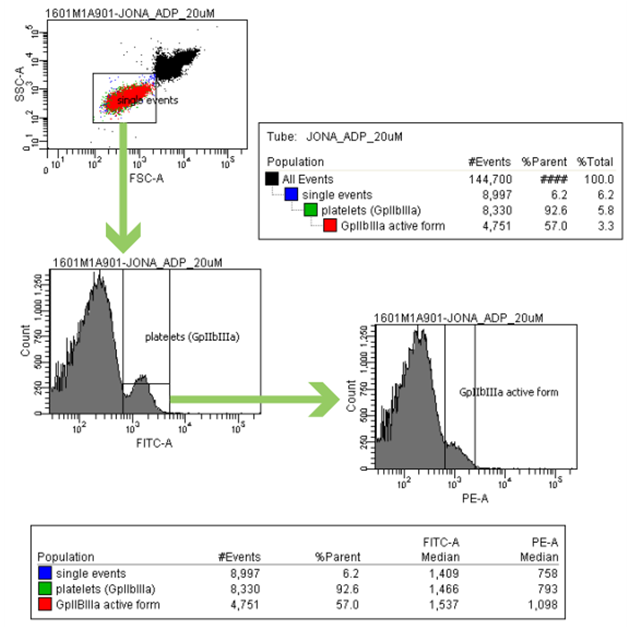

Supplement: Supplementary file 1 — Platelet population gating in LSRII using FACS/Diva version 6.0 software. Gating strategy for one of four antibodies where platelets positive for GpIIbIIIa (CD41/61) express active form of GpIIbIIIa after stimulation with ADP (20 μM). Population of platelets selected from diluted whole blood sample in two steps: firstly, based on forward-scatter (FFC) and side-scatter (SSC) characteristics; secondly, based on CD41/61 antigen positive expression. Finally, expression level of activation marker (GpIIbIIIa active form) measured in selected population of platelets. (TIF 132 kb) [file 13058_2018_1013_MOESM1_ESM.tif]
